# Swimming exercise counteracts metabolic and skeletal dysfunction in LDL receptor-deficient mice exposed to a high-fat diet

**DOI:** 10.1007/s00421-026-06396-2

**Published:** 2026-08-21

**Authors:** Roberto Conde Santos, Jose Antonio Dias Garcia, Jose Angelo Camilli, Wilson Romero Nakagaki, Luana Aparecida Chagas, Evelise Aline Soares, Silvia Graciela Ruginsk

**Affiliations:** 1 Faculty of Medicine, Federal University of Alfenas, Alfenas, MG Brazil; 2 University Jose do Rosario Vellano, Alfenas, MG Brazil; 3 Biology Institute, State University of Campinas, Campinas, SP Brazil; 4 Health Sciences, University of Western São Paulo, Presidente Prudente, SP Brazil; 5 Department of Physiological Sciences, Biomedical Sciences Institute, Federal University of Alfenas, 700 Gabriel Monteiro da Silva Street, Alfenas, MG 37130-001 Brazil

**Keywords:** Bone properties, Hyperlipidemia, Physical exercise, Lipid profile, Calcium, High-fat diet

## Abstract

**Purpose:**

Swimming exercise is widely recognized as an effective non-pharmacological intervention for the management of metabolic disorders; however, its impact on the interplay between dyslipidemia and skeletal integrity remains incompletely understood. This study investigated whether swimming exercise attenuates metabolic and bone alterations in low-density lipoprotein receptor-deficient (LDLr⁻/⁻) mice exposed to a high-fat diet (HFD).

**Methods:**

Mice were assigned to sedentary or exercise-trained groups and fed either standard chow or HFD. The training protocol consisted of daily swimming sessions (40 min/day) for 60 days.

**Results:**

LDLr⁻/⁻ mice displayed a marked hyperlipidemic phenotype, which was significantly attenuated by exercise, as evidenced by reduced total cholesterol and triglyceride concentrations and increased high-density lipoprotein levels. In parallel, exercise restored plasma calcium levels, suggesting improved mineral homeostasis. Sedentary LDLr⁻/⁻ mice exposed to HFD exhibited pronounced impairments in cortical and trabecular bone compartments, accompanied by reduced calcium levels and adverse lipid profiles. In contrast, exercise-trained animals, irrespective of genotype or diet, showed preservation of bone structural and mechanical properties. These findings indicate that swimming exercise mitigates both metabolic and skeletal dysfunction associated with LDL receptor deficiency and HFD exposure.

**Conclusion:**

The results support a role for aerobic exercise in maintaining systemic homeostasis under dyslipidemic conditions and highlight its potential as a non-pharmacological strategy to prevent the deleterious effects of combined genetic and dietary risk factors.

## Introduction

Hyperlipidemia encompasses a spectrum of disorders in lipid metabolism characterized by alterations in plasma lipoprotein concentrations, which contribute to the pathogenesis of chronic conditions, particularly those affecting the cardiovascular system (American Heart Association 2024). The most prevalent lipid abnormalities, including hypertriglyceridemia, hypercholesterolemia, reduced high-density lipoprotein (HDL) levels, and elevated low-density lipoprotein (LDL) concentrations, may occur either in isolation or in combination, and are strongly associated with cardiovascular risk. Specifically, increased LDL and total cholesterol (TC) levels are positively correlated with cardiovascular morbidity, whereas elevated HDL concentrations exert a protective effect (Abbasi et al. 2024).

Hyperlipidemias may be classified as primary, arising from genetic determinants, or secondary, resulting from underlying conditions such as chronic alcohol consumption, hypothyroidism, and obesity (Writing Committee 2016). In this context, LDL receptor-deficient (LDLr⁻/⁻) mice represent a well-established experimental model. When subjected to a high-fat diet (HFD), these animals develop phenotypic alterations that closely resemble human dyslipidemia, including elevated plasma TC and triglyceride (TG) levels, left ventricular hypertrophy, and atherosclerotic lesions (Krieger et al. 2006; Garcia et al. 2008). Given these systemic consequences, dyslipidemia constitutes a major public health concern, underscoring the importance of preventive and therapeutic strategies focused on lifestyle modification, including dietary regulation, regular physical activity, and maintenance of optimal body composition (American Heart Association 2024; Dawood et al. 2024; Bezner 2015; Manfredini et al. 2009).

Beyond its well-documented cardiovascular implications, emerging evidence indicates that hyperlipidemia also exerts deleterious effects on skeletal homeostasis. Recent studies have elucidated mechanisms linking elevated lipid levels to impaired bone metabolism, including the inhibition of osteoblast differentiation mediated by lipophagy, ultimately leading to reduced bone formation and remodeling capacity (He et al. 2024). Additionally, hyperlipidemia has been shown to disrupt the organization of type I collagen fibers, compromising cortical bone architecture and mechanical strength, particularly when associated with decreased bone mineral density (Ascenzi et al. 2014). These alterations are further exacerbated by increased oxidative stress within bone tissue (Sanbe et al. 2007) and impaired tendon-to-bone healing processes (Chung et al. 2016). Moreover, hyperlipidemia is frequently associated with hypovitaminosis D (Premaor and Furlanetto 2006), which may further compromise skeletal integrity, given the pivotal role of vitamin D in calcium homeostasis, collagen maturation, and the regulation of key osteogenic markers such as osteocalcin, osteopontin, and alkaline phosphatase (Bouillon et al. 1995).

Despite the important contributions of pharmacological interventions for management of hyperlipidemia-induced effects, non-pharmacological approaches, especially regular physical exercise, play a critical role in the prevention and management of chronic conditions, including bone loss. Aerobic exercise modalities, such as swimming, when performed in a structured and supervised manner, promote significant autonomic and hemodynamic adaptations that contribute to the maintenance of musculoskeletal homeostasis under increased physiological demand (Bezner 2015). Furthermore, physical activity may indirectly improve lipid profiles through reductions in adiposity (Manfredini et al. 2009), supporting its application not only in disease prevention but also in the rehabilitation of patients with osteoporosis and related disorders (Sinaki et al. 2010).

In light of this translational evidence, we hypothesized that swimming may mitigate metabolic and skeletal alterations in LDLr⁻/⁻ mice subjected to a high-fat diet (HFD). To test this hypothesis, LDLr⁻/⁻ mice, either sedentary or exercise-trained, and exposed or not to HFD, were evaluated for plasma lipid and calcium levels. In addition, femoral samples were subjected to biomechanical and morphometric analyses to assess bone structural properties.

## Methods and materials

### Animals and procedures

The experiments were carried out using male adult wild-type C57BL6 mice (as the control group) and homozygous mice for deletion of the LDL receptor gene (LDLr^−/−^). The animals were obtained from Jackson Laboratories (USA) and housed at the Animal Facility of the University Jose do Rosario Vellano (UNIFENAS). The animals were kept on ventilated shelves (Alesco^®^) with temperature control and a 12-hour light/dark cycle. After the acclimatization period, animals were randomly divided into four experimental groups of 12 individuals each, according to exercise (“sedentary” or “swimming”) and dietary protocols (“control or HFD”), with free access to the respective diet and tap water.

Swimming was used as the exercise protocol and was performed during 8 weeks (60 consecutive days) in individual tanks (30 cm x 15 cm x 15 cm), with water temperature kept between 32 and 36 °C. The training sessions always took place at the same time of day and were performed with progressive increase in both frequency and load, according to the following schedule, as previously employed by Guerino and coworkers (2008): first week: 3 weekly sessions of 10 min each, on alternate days, without load; second week: 4 weekly sessions, of 20 min each, with a load of 2.5% of the animal’s body weight; Third to eighth week: 5 weekly sessions, of 40 min each, with a load of 5% of the animal’s body weight. Training loads consisted of lead weights (up to 5% of the body mass) fixed with elastic straps to the animal’s thorax, and were placed immediately before swimming, in order to avoid movement impairments (Frateschi 2002). Thereafter, animals were placed in tanks, and monitored to avoid floating or leaning. The load value was adjusted weekly, according to body weight variation (Frateschi 2002; Evangelista et al. 2003). The animals belonging to the sedentary group were submitted to the aquatic environment for 1 min, following the same criteria as the trained groups, in order to mimic the stress condition. At the end of each swimming session, the animals were dried and returned to their cages.

For the dietary protocol, control groups were fed with commercial chow (Nuvital^®^), whereas HFD groups received a diet containing 20% total fat, 1.25% cholesterol and 0.5% of cholic acid, according to a modified AIN-93 protocol (Reeves et al. 1993; Table 1). All the experimental procedures were previously approved by the institutional Ethics Committee for Animal Use in Research of the University Jose do Rosario Vellano (UNIFENAS), under protocol number 189/2010A.

**Table 1:** Dietary composition of the high-fat chow

Component	Percentage (%)
Carbohydrates^1^	49
Casein (85% of protein)	21
Lipids^2^	20
Cellulose	5
Vitamin mix	1
Mineral mix	3
L-cystine	0,3
Choline bitartrate	0.25
Cholic acid	0.5
Tertbutylhydroquinone	0.0014
Total	100

Modified AIN-93, according to Reeves et al. (1993). ^1^Carbohydrates: starch = 54.25%, dextrinized starch = 26.64% and sucrose = 19.11%. ^2^Lipid: coconut fat = 70.25%, soybean oil = 23.75% and cholesterol = 6.25%. Caloric density = 2,89 Kcal/g

### Biochemical analyses

In order to analyze biochemical parameters, animals were euthanized with a lethal intraperitonial dose of ketamine (90 mg/Kg, Dopalen^®^, Brazil) and xylazine (10 mg/Kg, Rompun^®^, Brazil). Trunk blood was collected, centrifuged at 3,000 rpm, 4 °C for 20 min, and serum samples were used for determination of TG, TC, HDL and calcium concentrations using colorimetric enzymatic kits (Labtest^®^, Brazil) and a spectrophotometer (Gehaka G3410, Brazil) set at specific wavelengths (500, 540–570 nm), according to manufacturer’s instructions.

### Femur analyses

#### Bone dimensions

After euthanasia, femurs were removed and cleaned. Using a digital caliper and a magnifying glass, four measurements were estimated for each animal (Lammers et al. 1998): (1) femoral length (measured from the most proximal point of the femoral head to its most distant end); (2) proximal femur width (measured from the anterior point of the femoral head to the tip of the greater trochanter); (3) distal femur width (corresponding to the width of the condyle in the anteroposterior direction perpendicular to the length of the femur); (4) femoral diaphysis width (measured at the narrowest point of the mid-femur).

#### Mechanical test

The left femur was collected, cleaned, packed in gauze soaked in saline solution (NaCl 0.9%) and stored at − 20 °C until the day of the test. The following procedures were conducted according to previously described protocols (Nakagaki et al. 2011; Soares et al. 2012, 2015). The femur was mechanically tested until its complete fracture, in a three-point flexion procedure, at a speed of 0.05 mm/s (3 mm/min) (MTS, model TESTSTAR II), with a load of 100 Kgf. The top point and the two bottom brackets displayed a 0.5 mm radius at their ends. The distance between the two lower supports was 5 mm. Each femur was tested in the anteroposterior plane, with the anterior side facing up, supporting compression, and the posterior side facing down, supporting tension. The three-point bending test, employed in this study due to its suitability for long bones (Kruzic and Ritchie 2008), enables the assessment of both elastic and plastic deformation phases, ultimately determining fracture resistance (Alexandre et al. 2023; Cordey 2000).

**Fig. 1:**
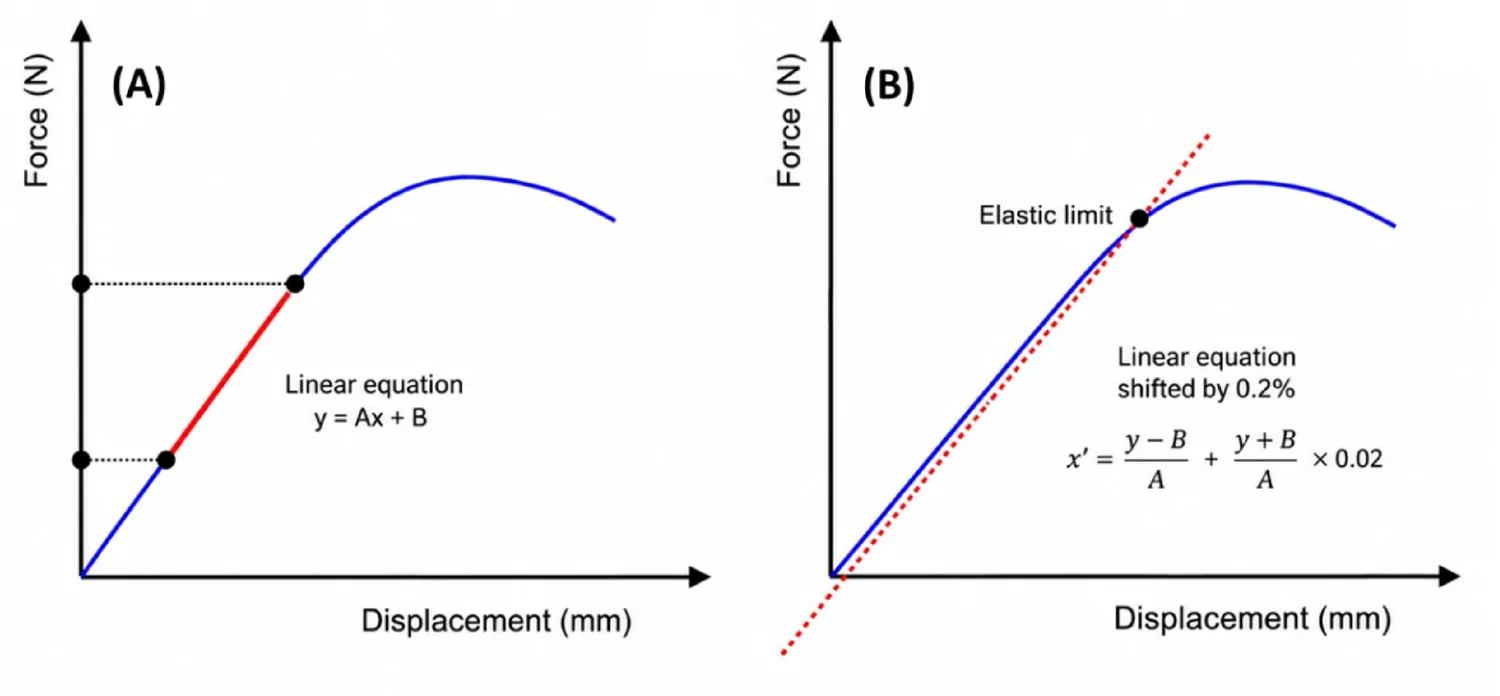
Determination of the equation of the line in the elastic region (phase) (**A**). Elastic limit determined exactly at the point where the curve and the new line intersect (**B**). In the equations, “y” is the variable representing force, “A” and “B” are constants, “x” is the variable representing displacements, and “x’” is the variable representing the new displacements on the line shifted by 0.2%

The data records were used for the measurement of structural properties, among them the maximum strength. The extrinsic stiffness was calculated as the slope of the most linear portion of the elastic region of the force-displacement curve, and was obtained at the point of the force-displacement curve called the elastic limit. In order to determine this point, the equation of the straight line of a linear section of the elastic phase was obtained (Fig. 1A), and, from this equation, a linear straight line displaced by 0.2% was obtained, which allowed the determination of the elastic limit, exactly at the point where this straight line and the force-displacement curve intersect (Fig. 1B).

After performing the three-point test on each specimen, the two ends of the rupture site were processed for the analysis of cross-sectional area of the diaphysis (cortical bone), whereas the distal ends of the femur were processed for histological procedure. For this purpose, 5 μm-thick sections were cut and stained with hematoxylin and eosin. The proximal extremities were photographed using a high-resolution digital camera with millimeter scale, with the aid of the NIS-Elements: Advanced Resect (USA) software. From the measurements obtained from each extremity, the average of each of the parameters was determined. The cross-sectional area parameters were used to calculate the diaphysial inertia moment, assuming that the diaphysis has an elliptical shape. The following equation was applied:

$$\:I=\:\frac{\pi\:}{64}\:[{ab}^{3}-\left(a-2t\right){\left(b-2t\right)}^{3}]$$

where “I” is the area moment of inertia; “a” is the width of the cross-sectional area in the mediolateral direction; “b” is the width of the cross-sectional area in the anteroposterior direction; and “t” is the average cortical bone thickness.

The mean cortical thickness was calculated from the measurements taken in each of the quadrants of the cortical area of the diaphysis. The material properties (maximum stress, elastic modulus and deformation) were calculated from these parameters using the equations below:

$$\:\sigma\:=\frac{strength\:.\:\:L\:.\:c}{4\:I}$$

$$\:\epsilon\:=\:\frac{12\:.\:\:c\:.\:\:d}{{L}^{2}}$$

$$\:E=\:\frac{stiffness\:.\:\:{L}^{3}}{48\:I}$$

where “σ” is the stress, “L” is the distance between the two lower supports, “c” is “b/2” (described previously), “ε” is the strain, “d” is the displacement and “E” is the elastic modulus. The force was either expressed as the maximum, or as the value corresponding to the elastic limit.

#### Trabecular and cortical area

For this analysis, six left femurs from each experimental group were used. For each femur, six histological sections were obtained, and, from each section, two fields were captured. Thus, a total of 72 randomly chosen fields were used. The samples were fixed in 10% buffered formaldehyde for 72 h at room temperature and decalcified in formic acid solution, formalin and sodium citrate for 35 days. Then, the samples were processed using routine histological techniques and embedded in paraffin. 5 μm-thick cross-sections were stained with hematoxylin and eosin. Next, the measurement of the area and thickness of the bone trabeculae and cortices in the distal epiphysis and diaphysis was performed (NIS-Elements, Advanced Research, USA) under 20 X magnification (Nikon Eclipse E400, Nikon DXM 1200 C camera).

### Statistical analysis

The results were expressed as mean ± standard error of the mean (SEM) and analyzed using two-way Analysis of Variance (ANOVA), followed by Tukey’s post-test. The level of significance adopted was 5% (bicaudal).

## Results

Table 2 shows that trained WH animals exhibited a significant decrease in water intake, when compared to the respective sedentary group (*p* < 0.001). There was also a slight increase in food intake in trained LH animals, when compared to the sedentary mates (*p* < 0.05). Therefore, we could not detect any effect of diet or genetic manipulation on water and food intakes, but, as expected, body mass gain was significantly increased in WH groups (*p* < 0.001), regardless of exercise, being this effect completely abolished in LDLr^−/−^ animals.

**Fig. 2:**
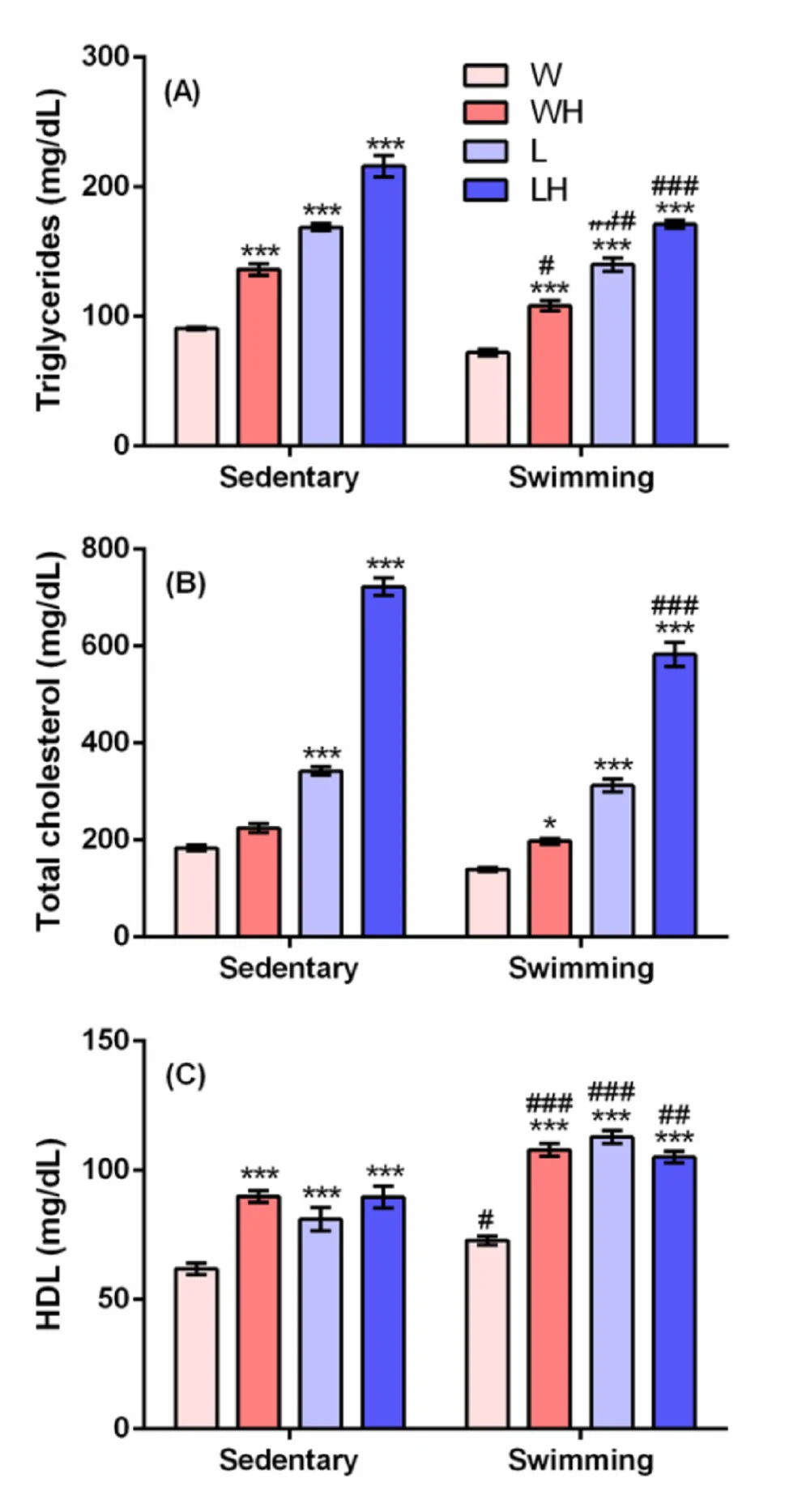
Plasma concentrations of triglycerides (**A**), total cholesterol (**B**) and HDL (**C**) in wild-type (W) and LDLr-/- (L) sedentary and trained mice fed with standard or high-fat diet (H). Results are expressed as mean ± SEM and were analyzed by Two-way ANOVA, followed by Tukey’s post-test. *p < 0.05 and *** *p* < 0.001 versus the respective wild-type group; # *p* < 0.05, ## *p* < 0.01 and ### *p* < 0.001 versus the respective sedentary group

According to Fig. 2A, TG were elevated in sedentary WH group (90.88 ± 1.04 *versus* 136.36 ± 4.41 mg/ dL, *p* < 0.001), and also in LDLr^−/−^ groups, regardless of diet (L groups: 90.88 ± 1.04 *versus* 168.83 ± 2.81 mg/dL, *p* < 0.001; LH group: 90.88 ± 1.04 *versus* 216.07 ± 8.16 mg/dL, *p* < 0.001). The same response was observed in the groups submitted to swimming. However, the trained WH, L and LH groups showed attenuated plasma TG compared to the respective sedentary groups (WH groups: 108.07 ± 4.02 *versus* 136.36 ± 4.41 mg/dL, *p* < 0.001; L groups: 140.08 ± 5.35 *versus* 168.83 ± 2.81 mg/dL, *p* < 0.001; LH groups: 171.21 ± 3.11 *versus* 216, 07 ± 8.16 mg/dL, *p* < 0.001).

Sedentary LDLr^−/−^ animals, regardless of diet, also showed increased concentrations of TC, when compared to the respective wild-type group (L groups: 342.32 ± 8.78 *versus* 183.93 ± 5.51 mg/dL, *p* < 0.001; LH groups: 722.31 ± 17.77 *versus* 183.93 ± 5.51 mg/dL, *p* < 0.001, Fig. 2B). Again, the same effect was observed in the trained groups (L groups: 312.14 ± 13.25 *versus* 139.36 ± 4.42 mg/dL, *p* < 0.001; LH groups: 582.69 ± 24.21 *versus* 139, 36 ± 4.42 mg/dL, *p* < 0.001). Swimming only prevented the diet-induced increase in CT levels in the LH group (722.31 ± 17.77 *versus* 582.69 ± 24.21 mg/dL, *p* < 0.001). Additionally, physical exercise significantly increased TC levels in WH animals (197.66 ± 5.76 versus 139.36 ± 4.42 mg/dL, *p* < 0.05).

Finally, sedentary WH, L and LH groups showed increased HDL levels (61.92 ± 2.23 *versus* 89.87 ± 2.14 mg/dL, *p* < 0.001; 61.92 ± 2.23 *versus* 81.07 ± 4.54 mg/dL, *p* < 0.001; 61.92 ± 2.23 *versus* 89.68 ± 4.20 mg/dL, *p* < 0.001, respectively; Fig. 2C). This effect was even more pronounced in the groups submitted to swimming, including the control group. Thus, the beneficial effect of physical activity in increasing HDL concentrations also occurred in sedentary groups, regardless of the diet (W groups: 61.92 ± 2.23 *versus* 72.88 ± 1.69 mg/dL, *p* < 0.05; WH groups: 89.87 ± 2.14 *versus* 107.86 ± 2.57 mg/dL, *p* < 0.001; L groups: 81.07 ± 4.54 *versus* 112.87 ± 2.58 mg/dL, *p* < 0.001; LH groups: 89.68 ± 4.20 *versus* 105.11 ± 2.21 mg/dL, *p* < 0.001).

**Fig. 3:**
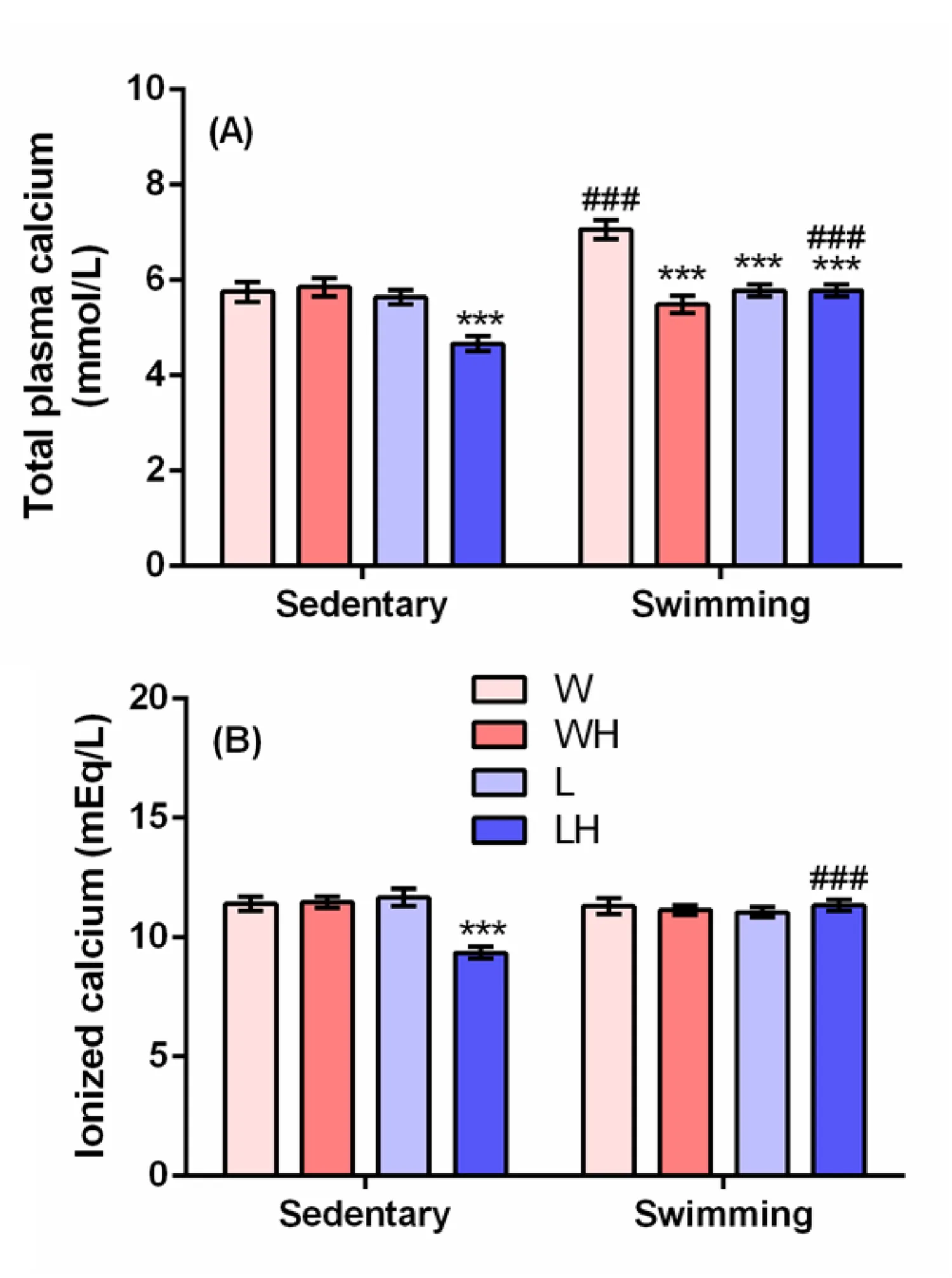
Plasma concentrations of total (**A**) and ionized calcium (**B**) in wild-type (W) and LDLr-/- (L) sedentary and trained mice fed with standard or high-fat diet (H). Results are expressed as mean ± SEM and were analyzed by Two-way ANOVA, followed by Tukey's post-test. *** *p* < 0.001 versus the respective wild-type group and ### *p* < 0.001 versus the respective sedentary group

Figure 3 represents total (A) and ionized (B) calcium concentrations in all experimental groups. Sedentary LH animals showed a significant reduction in total calcium levels, when compared to the respective W group (5.75 ± 0.20 *versus* 4.66 ± 0.15 mmol/L, *p* < 0.001), being this effect completely reversed by physical exercise (4.66 ± 0.15 *versus* 5.78 ± 0.12 mmol/L, *p* < 0.001). Furthermore, swimming increased total calcium concentrations in W group (5.75 ± 0.20 *versus* 7.05 ± 0.19 mmol/L, *p* < 0.001) and decreased the same parameter in WH and L groups (SH groups: 5.49 ± 0.17 *versus* 7.05 ± 0.19 mmol/L, *p* < 0.001; L groups: 5.78 ± 0.13 *versus* 7.05 ± 0.19 mmol/L, *p* < 0.001). A significant reduction of ionized calcium levels was also found in sedentary LH animals (11.41 ± 0.30 *versus* 9.36 ± 0.25 mEq/L, *p* < 0.001). Again, swimming completely reversed this effect (9.36 ± 0.25 *versus* 11.34 ± 0.24 mEq/L, *p* < 0.001).

Regarding bone structural and functional properties, Table 3 shows that both the sedentary LH group and trained W animals showed decreased femoral length when compared to the W group (*p* < 0.001 or *p* < 0.05, respectively). The width of the distal femur, in turn, was significantly reduced in the sedentary LH group, compared to the respective W group (*p* < 0.05). No significant changes were observed in the width of the proximal femur. Among the groups submitted to the swimming protocol, we only observed a reduction in the width of the distal femur in the L group, compared to the respective W group (*p* < 0.01). Among the sedentary animals, all LDLr knockout mice showed reduced width of the femoral diaphase, regardless of diet (*p* < 0.001). Swimming not only reversed this effect for the LH group, but also increased the diaphase width in L animals, in relation to the respective sedentary L group (*p* < 0.05).

**Fig. 4:**
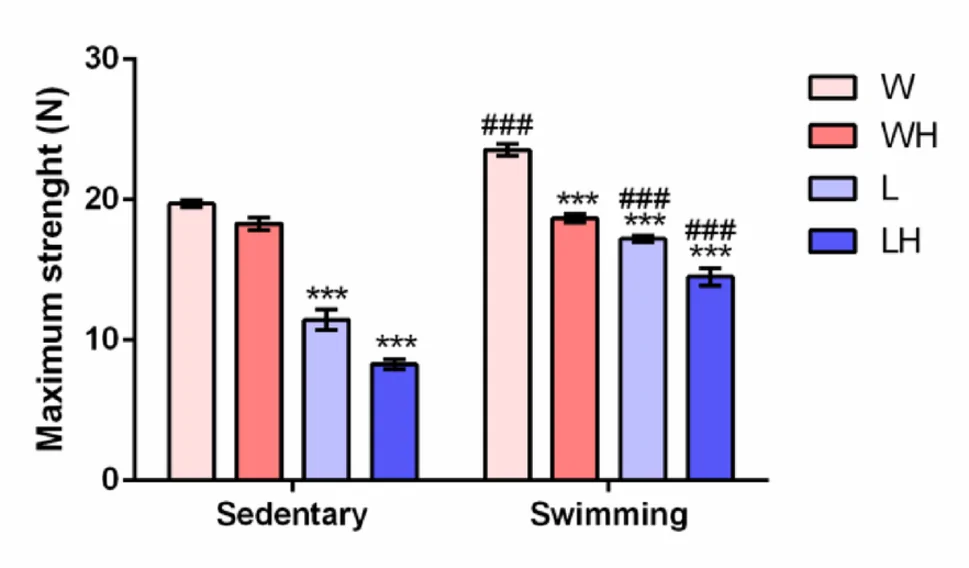
Maximum strength in wild type (W) and LDLr-/- (L) sedentary and trained mice fed with standard or high-fat diet (H). Results are expressed as mean ± SEM and were analyzed by Two-way ANOVA, followed by Tukey’s post-test. *** *p* < 0.001 versus the respective wild-type group and ### *p* < 0.001 versus the respective sedentary group

We also analyzed bone performance in the mechanical test (Fig. 4), and observed, among sedentary animals, a very clear effect of genetic manipulation, associated or not with diet, in reducing maximum strength (L groups: 19.71 ± 0.26 *versus* 11.44 ± 0.70 N, *p* < 0.001; LH groups: 19.71 ± 0.26 *versus* 8.27 ± 0.37 N, *p* < 0.001). In the trained groups, the beneficial effect of swimming was already evident in the control group (19.71 ± 0.26 *versus* 23.53 ± 0.43 N, *p* < 0.001). Although exercise did not modify bone performance among WH mice, we observed that swimming attenuated (but did not prevent) the effects of genetic manipulation, associated or not with diet, on maximum strength (L groups: 11.44 ± 0.70 *versus* 17.20 ± 0.22 N, *p* < 0.001; LH groups: 8.27 ± 0.37 *versus* 14.50 ± 0.62 N, *p* < 0.001). Thus, the harmful bone outcomes induced by increased serum lipid concentrations remain clear in the mechanical resistance test. Exercise, in turn, partially reversed these effects.

**Fig. 5:**
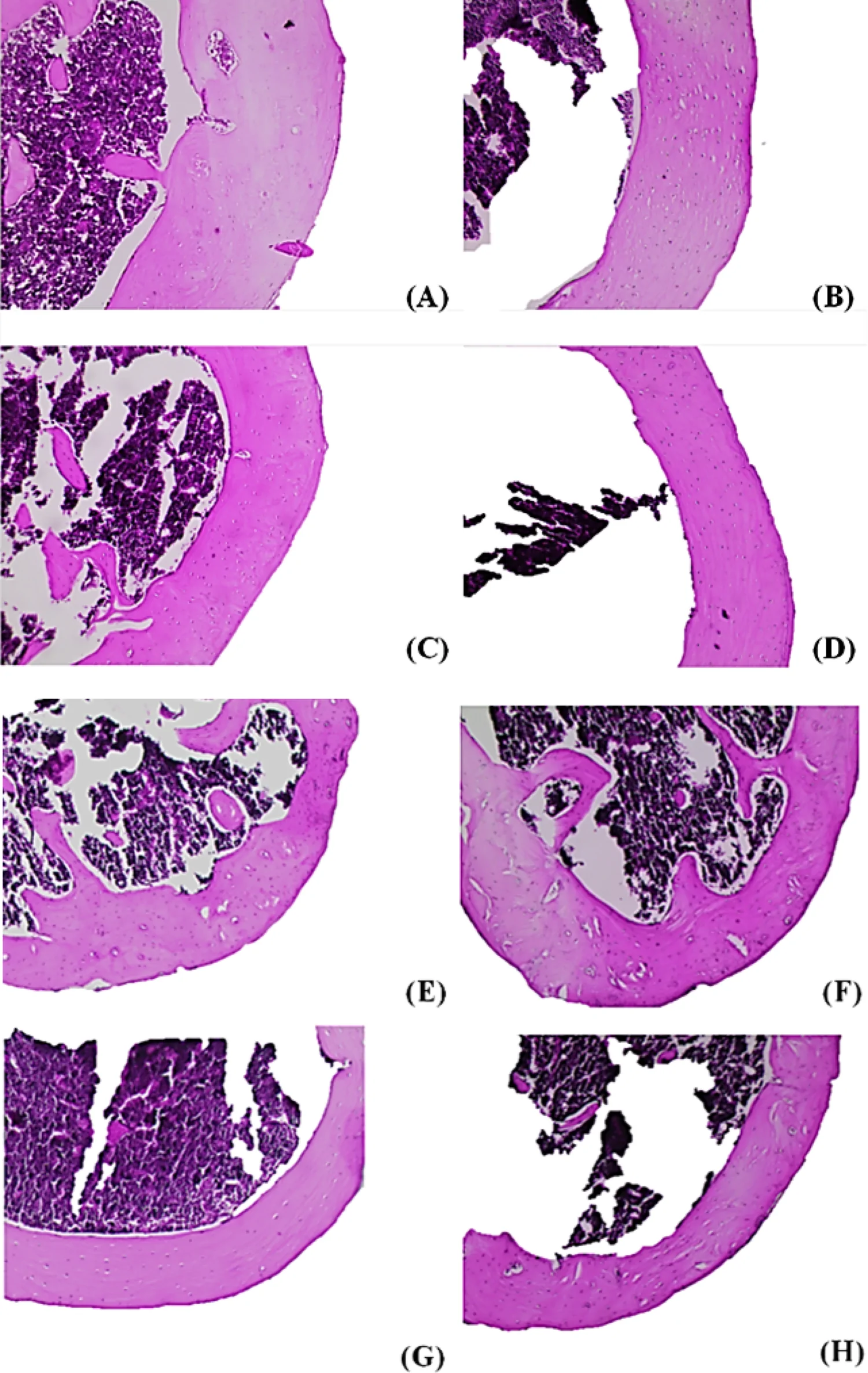
Representative photomicrographs of cross-sections of the femoral diaphases. Wild type trained (**A**), wild type sedentary (**B**), wild type trained fed with high-fat diet (C), wild type sedentary fed with high-fat diet (D), LDLr^−/−^ trained (E), LDLr^−/−^ sedentary (F), LDLr^−/−^ trained fed with high-fat diet (G) and LDLr^−/−^ sedentary fed with high-fat diet (H). Images were captured under an optical microscope with 4x magnification and stained with hematoxylin and eosin

**Fig. 6:**
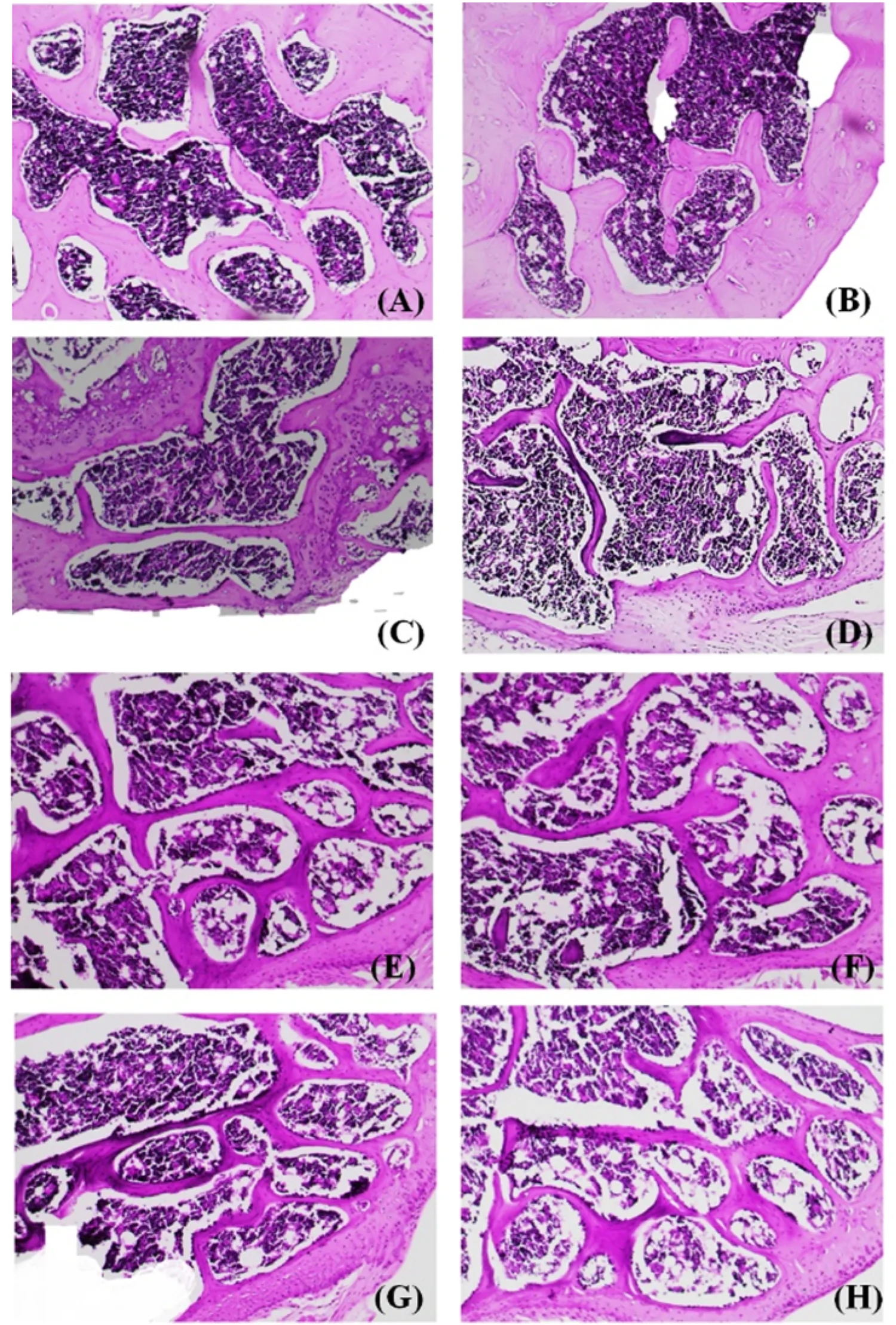
Representative photomicrographs of cross-sections of the femoral epiphyses. Wild type trained (**A**), wild type sedentary (**B**), wild type trained fed with high-fat diet (**C**), wild type sedentary fed with high-fat diet (**D**), LDLr^−/−^ trained (**E**), LDLr^−/−^ sedentary (**F**), LDLr^−/−^ trained fed with high-fat diet (**G**) and LDLr^−/−^ sedentary fed with high-fat diet (**H**). Images were captured under an optical microscope with 4x magnification and stained with hematoxylin and eosin

In parallel to maximum strength, bone physical characteristics, cortical area, cortical thickness and trabecular area provide important information regarding fracture resistance. The morphometric characteristics (trabecular/cortical area, trabecular/cortical thickness) and biomechanical properties (displacement, extrinsic stiffness, maximum tension, modulus of elasticity and deformation) of the different experimental groups are shown in Table 4 and illustrated by representative photomicrographs of Figs. 5 and 6, which demonstrate, respectively, femoral diaphases and epiphyses.

According to Table 4, there was a clear and significant reduction in the trabecular thickness and area in the sedentary LH group, when compared to the respective W group (*p* < 0.001). Although the thickness was not reestablished by swimming, the trabecular area was completely recovered in trained LH animals (*p* < 0.001). The cortical area was not altered by diet or exercise, despite the significant reduction in cortical thickness observed in WH (*p* < 0.001), L (*p* < 0.01) and LH mice (*p* < 0.001). While the effects of exercise in morphometric characteristics were not so prominent, particularly in the cortical bone, the protective actions of physical training on biomechanical properties were very clear. The displacement was unaltered in the sedentary groups, and significantly reduced in trained WH, L and LH animals (*p* < 0.01). There was also an effect of exercise in increasing displacement in the W mice, when compared to the sedentary mates (*p* < 0.01). Furthermore, extrinsic stiffness was significantly reduced in the sedentary WH, L and LH groups (*p* < 0.001), and swimming only partially reversed this effect. The same profile was observed for maximum tension, with a positive effect of physical exercise being detected in the control group (*p* < 0.001). The elastic modulus was also reduced by the dietary intervention, either combined or not with LDLr deletion. This effect was partially reverted by physical exercise only in the W and LH groups (*p* < 0.001). Finally, deformation was reduced in all sedentary groups, compared to the respective W group (*p* < 0.001), and swimming significantly restored these values in all experimental groups, including the W group.

**Table 2:** Mean fluid intake (mL/day), mean food consumption (g/day) and body mass gain (g) in in wild-type (W) and LDLr^-/-^ (L) sedentary and trained mice fed with standard or high-fat diet (H)

Variable	Group	W		WH		L		LH	
		Mean	SEM	Mean	SEM	Mean	SEM	Mean	**SEM**
Mean fluid intake	Sedentary	8.10	0.26	8.87	0.15	8.45	0.25	7.92	0.21
	Swimming	8.13	0.11	7.35^###^	0.15	8.02	0.28	8.60	0.27
Mean food consumption	Sedentary	6.47	0.15	6.82	0.17	6.97	0.19	6.77	0.30
	Swimming	6.92	0.09	7.37	0.11	7.32	0.29	7.67^#^	0.21
Body mass gain	Sedentary	6.22	0.31	7.90^***^	0.12	6.65	0.15	7.00	0.31
	Swimming	6.04	0.28	7.67^***^	0.21	6.70	0.24	6.72	0.18

Results are expressed as mean ± standard error of the mean (SEM) and were analyzed by Two-way Analysis of Variance, followed by Tukey’s post-test. *** *p*<0.001 versus respective wild-type group (same line); # *p*<0.05 and ### *p*<0.001 versus the respective sedentary group (same column)

**Table 3:** Femoral dimensions (in mm) in wild-type (W) and LDLr^-/-^ (L) sedentary and trained mice fed with standard or high-fat diet (H)

Variable	Group	W		WH		L		LH	
		Mean	SEM	Mean	SEM	Mean	SEM	Mean	**SEM**
Femoral length	Sedentary	13.00	0.21	12.08	0.26	12.00	0.27	11.33^***^	0.25
	Swimming	11.75^#^	0.37	11.75	0.28	11.41	0.28	11.08	0.39
Proximal femur width	Sedentary	3.41	0.15	3.57	0.13	3.05	0.13	3.10	0.15
	Swimming	3.57	0.15	3.38	0.13	3.26	0.19	3.13	0.15
Distal femur width	Sedentary	3.49	0.10	3.27	0.17	3.21	0.08	3.07^*^	0.07
	Swimming	3.66	0.13	3.28	0.07	3.11^**^	0.06	3.30	0.05
Femoral diaphase width	Sedentary	2.22	0.07	2.13	0.01	1.89^***^	0.06	1.86^***^	0.06
	Swimming	2.15	0.01	2.15	0.01	2.10^#^	0.06	2.03	0.06

Results are expressed as mean ± standard error of the mean (SEM) and were analyzed by Two-way Analysis of Variance, followed by Tukey’s post-test. * *p*<0.05, ** *p*<0.01 and *** *p*<0.001 *versus* the respective wild-type (same line); # *p*<0.05 *versus* the respective sedentary group (same column)

**Table 4:** Morphometric characteristics (trabecular/cortical area: mm^2^, and trabecular/cortical thickness: mm) and biomechanical properties (displacement: mm, extrinsic stiffness: N/mm, maximum stress: MPa, Elasticity modulus: Mpa, and deformation: Mpa) of femurs obtained from wild-type (W) and LDLr^-/-^ (L) sedentary and trained mice fed with standard or high-fat diet (H)

Variable	Group	W		WH		L		LH	
		Mean	SEM	Mean	SEM	Mean	SEM	Mean	SEM
Trabecular area	Sedentary	3.70	0.13	3.35	1.00	3.33	0.15	2.21^***^	0.12
	Swimming	3.71	0.12	3.42	0.13	3.77	0.12	3.30^###^	0.11
Trabecular thickness	Sedentary	0.20	0.01	0.19	0.006	0.13^***^	0.013	0.13^***^	0.01
	Swimming	0.23	0.01	0.21	0.009	0.18^**##^	0.007	0.13^***^	0.01
Cortical area	Sedentary	4.85	0.07	4.76	0.05	4.63	0.10	4.56	0.14
	Swimming	4.78	0.05	4.86	0.12	4.60	0.11	4.43	0.07
Cortical thickness	Sedentary	0.96	0.02	0.87	0.02	0.80^**^	0.02	0.74^***^	0.03
	Swimming	1.01	0.04	0.80^***^	0.04	0.83^**^	0.03	0.80^***^	0.03
Displacement	Sedentary	0.32	0.02	0.25	0.02	0.27	0.02	0.30	0.02
	Swimming	0.42^##^	0.01	0.31^**^	0.01	0.31^**^	0.02	0.31^**^	0.03
Extrinsic stiffness	Sedentary	132.75	4.05	84.66^***^	1.76	76.33^***^	1.40	70.66^***^	1.32
	Swimming	137.08	1.62	92.58^***#^	1.11	98.08^***###^	1.60	82.08^***##^	2.93
Maximum stress	Sedentary	16.15	0.35	12.40^***^	0.54	10.80^***^	0.23	10.73^***^	0.34
	Swimming	20.42^###^	0.56	14.16^***##^	0.41	13.40^***###^	0.29	14.44^***###^	0.31
Elasticity modulus	Sedentary	116.81	3.87	99.91^**^	1.81	66.73^***^	4.09	69.00^***^	1.77
	Swimming	169.70^***###^	4.21	101.84^***^	1.53	77.47^***^	3.93	87.11^***###^	2.08
Deformation	Sedentary	0.17	0.01	0.05^***^	0.007	0.08^***^	0.010	0.09^***^	0.01
	Swimming	0.25^***###^	0.01	0.11^***###^	0.005	0.17^***###^	0.014	0.14^***#^	0.01

Results are expressed as mean ± standard error of the mean (SEM) and were analyzed by Two-way Analysis of Variance, followed by Tukey’s post-test. * *p*<0.05. ** *p*<0.01 and *** *p*<0.001 *versus* the respective wild-type group (same line); # *p*<0.05. ## *p*<0.01 and ### *p*<0.001 *versus* the respective sedentary group (same column)

## Discussion

In the present study, we investigated the interplay between aerobic exercise and HFD exposure on metabolic, biomechanical, and morphometric parameters in LDLr-deficient mice. The findings demonstrate that knockout animals developed a hyperlipidemic phenotype, whereas swimming exercise, irrespective of dietary condition, effectively reduced TC and triglyceride TG levels, increased HDL concentrations, and restored plasma calcium homeostasis. Notably, femoral analyses revealed pronounced impairments in cortical and trabecular compartments in sedentary LDLr⁻/⁻ mice exposed to HFD, occurring in parallel with dyslipidemia and hypocalcemia. In contrast, both wild-type and LDLr⁻/⁻ mice subjected to exercise training exhibited elevated HDL levels, which may have conferred protection against structural bone deterioration. Collectively, these findings support the central hypothesis that swimming mitigates the metabolic and skeletal disturbances induced by LDLr deficiency, either alone or in combination with HFD.

In the present study, minor variations in food and fluid intake were detected in response to exercise, reflecting physiological ranges for murine models. With respect to nutritional status, all experimental groups exhibited body mass gain over the course of the study. HFD promoted a significant increase in body mass in wild-type animals, an effect that was not observed in LDLr⁻/⁻ mice, suggesting genotype-specific metabolic adaptations. These findings corroborate previously published data showing that LDLr knockout mice exhibit decreased susceptibility to Western-type diet-induced obesity (Ngai et al. 2010), possibly due to an increased basal thermogenic activity and a disrupted response to leptin administration. However, the literature also reported increased susceptibility of LDLr⁻/⁻ mice to diet-induced weight gain (Schreyer et al. 2002; Sithu et al. 2017), which is aligned, under a translational perspective, with the higher prevalence of overweight and obesity observed in human familial hypercholesterolemia (Elshorbag et al. 2025).

In this regard, current international guidelines for cardiovascular risk management emphasize lifestyle modification, particularly regular physical activity, as the first-line approach for hyperlipidemia control, with pharmacological interventions reserved for refractory cases (American Heart Association 2024). In agreement with this paradigm, the present results demonstrate that exercise training attenuated HFD-induced dyslipidemia even in LDLr⁻/⁻ mice, which inherently exhibit hypercholesterolemia under standard dietary conditions. These findings are consistent with previous reports showing that swimming reduces atherosclerotic lesion development in ApoE-deficient mice (Zhang et al. 2021), as well as with both clinical and experimental animal studies demonstrating that chronic exercise improves lipid metabolism by increasing HDL concentration and functionality, reducing plasma TG, enhancing reverse cholesterol transport, and promoting fatty acid oxidation, thereby decreasing basal circulating free fatty acid availability (Rahmati-Ahmadabad et al. 2019). Together, these data reinforce the metabolic benefits of aerobic exercise, even in genetically predisposed models.

The present study also evaluated total and ionized plasma calcium concentrations, providing additional insight into the systemic effects of hyperlipidemia. In mammals, approximately 99% of total body calcium is stored in the skeleton, with only a small fraction present in circulation in protein-bound, ionized, or complexed forms. Here, sedentary LDLr⁻/⁻ mice exposed to HFD exhibited reductions in both total and ionized calcium, whereas exercise restored or even elevated these parameters across most experimental groups. These findings are consistent with previous studies reporting reduced calcium levels in LDLr-deficient mice (Soares et al. 2012) and further support the notion that hyperlipidemia adversely affects mineral homeostasis.

Mechanistically, dyslipidemia has been associated with impaired bone remodeling through increased osteoclastic activity and suppression of osteoblast differentiation, ultimately disrupting the balance between bone formation and resorption (He et al. 2024; Nuzzo et al. 2009; Luegmayr et al. 2004). Also, there is evidence of consistent association between reduced bone mineral density, vascular calcification, and atherosclerosis, although this relationship appears to be complex and multifactorial (Persy and D’Haese 2009). Within this context, atherosclerotic calcification is an active process of vascular wall biomineralization that is frequently observed in individuals with hyperlipidemia and other metabolic disorders (Abedin et al. 2004). Current evidence suggests that vascular calcification and bone loss share common pathophysiological mechanisms, although there is no definitive evidence that the calcium deposited in the arterial wall is directly derived from bone tissue (Collin-Osdoby 2004; Abedin et al. 2004; Persy and D’Haese 2009).

In the present study, swimming also improved total and ionized calcium levels in LDLr-deficient mice fed with a HFD. In this regard, experimental studies in rodents indicate that chronic exercise improves calcium homeostasis by enhancing intestinal calcium absorption, increasing calcium balance, modulating calciotropic hormones, and promoting skeletal calcium retention (LeBlanc et al. 1983; Yeh and Aloia 1990). Although serum ionized calcium is tightly regulated and is not consistently increased by exercise, these adaptations may contribute to the maintenance or restoration of mineral homeostasis under conditions of impaired calcium metabolism.

Morphometric analyses further demonstrated that the combined effects of LDLr deletion and HFD negatively impacted femoral geometry, particularly diaphyseal width, an effect that was attenuated by exercise. These structural alterations are functionally relevant, as bone geometry is a key determinant of mechanical competence. Consistent with previous reports (Soares et al. 2012), femurs from LDLr⁻/⁻ mice exposed to HFD exhibited reduced maximal strength compared to controls. In addition, in their study, parameters such as cortical area, cortical thickness, and trabecular area were markedly compromised under hyperlipidemic conditions, particularly when genetic and dietary factors were combined, mostly corroborating the present findings. Futhermore, exercise exerted modest protective effects on these morphometric indices, as also demonstrated by the present results.

In contrast, biomechanical analyses revealed that most of structural and material properties were significantly impaired by HFD, regardless of genotype. These results corroborate previous findings of our group showing that hyperlipidic diet functions as a risk factor for bone integrity, inducing reduced resistance and increased susceptibility to fractures, being these outcomes reversed by simvastatin administration (Soares et al. 2015). The observed reductions in bone rigidity may be attributed to decreased mineralization, matrix degradation, and alterations in collagen organization, which collectively influence the elastic modulus (Ferretti et al. 2001). Furthermore, hyperlipidemia-induced skeletal alterations have also been linked to reduced nitric oxide (NO) bioavailability, a key regulator of osteoblast differentiation and bone formation (Marte and Santos 2007). Thus, the deleterious effects of dyslipidemia on bone tissue appear to involve both metabolic and signaling pathways that converge on impaired remodeling dynamics. Importantly, exercise training restored the majority of these parameters, highlighting again its role in preserving bone mechanical integrity.

To our knowledge, this is among the first studies to simultaneously demonstrate that aerobic exercise improves lipid metabolism, restores calcium homeostasis, and preserves both bone morphology and mechanical competence in LDLr-deficient mice exposed to HFD. Considering that familial hypercholesterolemia and diet-induced dyslipidemia are increasingly recognized as conditions associated with impaired bone remodeling and vascular calcification, these results reinforce the concept of a functional bone-vascular axis in which metabolic disturbances simultaneously affect cardiovascular and skeletal health. Although the molecular mechanisms underlying these interactions remain to be fully elucidated, the present study expands current knowledge by demonstrating that regular aerobic exercise may counteract both metabolic and skeletal consequences of LDL receptor deficiency. These findings support physical exercise as a promising non-pharmacological strategy to preserve mineral homeostasis and bone quality in individuals at high cardiovascular risk.

## Conclusions

Taken together, the present findings demonstrate that the combination of LDLr deficiency and HFD promotes significant metabolic and skeletal dysfunction, encompassing biochemical, structural, and functional alterations. Notably, swimming exercise effectively counteracted most of these deleterious effects, supporting its role as a non-pharmacological strategy in the management of hyperlipidemia and its systemic complications. These results further underscore the importance of integrating regular physical activity with dietary interventions to mitigate the multifactorial impact of dyslipidemia on both cardiovascular and skeletal health.
